# A fast workflow to explore active enzymes from environmental samples through functional metagenomics

**DOI:** 10.1007/s00253-026-13805-1

**Published:** 2026-03-25

**Authors:** Arief Muammar, Endah Retnaningrum, Budi Setiadi Daryono, Irfan Dwidya Prijambada, Yuki Yashima, Clemens Peterbauer

**Affiliations:** 1 Food Biotechnology Laboratory, Department of Biotechnology and Food Sciences, BOKU University, Muthgasse 11, 1190 Vienna, Austria; 2 Department of Tropical Biology, Faculty of Biology, Universitas Gadjah Mada, Jalan Teknika Selatan, Sinduadi, Mlati, Sleman, 55281 Special Region of Yogyakarta Indonesia; 3 Faculty of Agriculture, Universitas Gadjah Mada, Jalan Flora, Bulaksumur, Sleman, 55281 Special Region of Yogyakarta Indonesia; 4 Department of Biomaterial Sciences, Graduate School of Agricultural and Life Sciences, The University of Tokyo, 1-1-1 Yayoi, Bunkyo-ku, Tokyo, 113-8657 Japan

**Keywords:** Functional metagenomic, Multiplex PCR, Rolling circle amplification, Cellulase

## Abstract

**Abstract:**

Functional metagenomics has emerged as an effective tool for discovering novel enzymes directly from environmental samples, overcoming the limitations of traditional culture-based methods. In this study, we used a functional metagenomic approach on stool samples from *Axis kuhlii*, an endemic deer species from Indonesia, to identify active cellulases. We created an efficient workflow for expression of metagenomic sequences directly in *Komagatella phaffii* by combining metagenomic sequencing to investigate enzyme diversity, multiplex PCR to build a genes library, and rolling circle amplification (RCA) to streamline the cloning process, eliminating the need for intermediate *Escherichia coli* transformation and propagation steps. Furthermore, a semi-high-throughput screening method was used to evaluate multiple samples at once, allowing for the rapid identification of active enzymes. Using this approach, we discovered five endoglucanases and three β-glucosidases with confirmed enzyme activity. This study shows that functional metagenomics can bridge the gap between computational predictions and experimental validation, providing a reliable platform for enzyme discovery and characterization from complex environmental microbiomes.

**Key points:**

• *We established K. phaffii expression of metagenomic sequences via multiplex PCR and RCA.*

• *This approach links metagenomic and activity screening to enable enzyme discovery.*

• *Eight active cellulases were obtained from environmental samples through this approach.*

**Supplementary Information:**

The online version contains supplementary material available at 10.1007/s00253-026-13805-1.

## Introduction

Metagenomics is a culture-independent approach that extracts and analyzes environmental DNA (eDNA) from microbial communities. This method is especially important because traditional microbiological techniques can only cultivate around 1% of prokaryotic microorganisms, leaving the vast majority of microbial diversity unexplored. By eliminating the need for cultivation, metagenomics enables researchers to access the genetic material of unculturable microorganisms, which account for roughly 99% of microbial diversity (Culligan et al. 2013). This metagenomic approach has transformed biotechnology by allowing the discovery of novel genes and enzymes with distinct properties.

Robinson et al. (2021) classified metagenomic enzyme discovery into three groups: De novo discovery: identifying entirely new enzyme types with no characterized members; Reference-based discovery: characterizing new reactions within known protein families; and Enzyme expansion: focusing on extending the properties of known enzyme classes. The term Enzyme expansion rather than enzyme discovery was used because this approach is mostly used to improve characterized enzymes with different substrate preferences. The de novo discovery represents the most challenging and novel discoveries, targeting entirely uncharacterized enzyme families. This approach is mostly used in cultivated microbes, to learn more about novel substrates, cofactors, and reactions. The reference-based discovery involves finding new functions within known families. This approach provides high-throughput annotation of eDNA datasets where some reference points are available.

Functional metagenomics, as a subset of metagenomic enzyme discovery, not only allows researchers to learn about the diversity and abundance of novel genes or enzymes, but also to obtain and produce functional novel proteins. Berini et al. (2017) reviewed 332 metagenome-sourced enzymes from 2014 to 2017, but only few have been optimized for industrial applications, leaving room for further development. The limited optimization of metagenome-sourced enzymes for industrial applications is due to technical, economic, and practical challenges, as well as a prioritization of discovery over application. This opens up significant opportunities for further development and industrial exploitation of these enzymes (Seo et al. 2014).

Lignocellulose has become a popular target for metagenomic enzyme discovery due to its abundance, complexity, and industrial potential. The metagenome sample we used was stool from an endemic and geographically isolated deer species, *Axis kuhlii*. It is native to the small island of Bawean in Indonesia and is classified as critically endangered. We compared the metagenomic sequences with known protein families (reference-based discovery) and used multiplex PCR and rolling circle amplification to prepare libraries specific for different cellulase activities. We then screened the libraries for endoglucanase and β-glucosidase activity, identifying several functional enzymes.

## Material and methods

### Cellulase diversity by metagenomic sequencing

Stool samples were collected from free-ranging *Axis kuhlii* from Bawean Island Wildlife Reserve (samples 1–3) as well as from captive animals in two different facilities within the wildlife reserve (samples 4–6 and 7–9). Metagenomic DNA from the stool samples was extracted using the Quick-DNA™ HMW MagBead Kit from Zymo Research (Irvine, CA). A DNA library was prepared with the DNA library preparation kit and sequenced using a Next Generation Sequencing (NGS) approach on a Promethion sequencer (Oxford Nanopore Technologies, Oxford, UK). Basecalling was done using Guppy v2.2.3, and the raw reads were exported as fastq files and subsequently concatenated by sample. A quality control analysis of raw reads was performed using longQC.py v1.2.1 (Fukasawa et al. 2020), and the results were manually inspected. The raw reads were assembled using Canu v2.2 (parameters: -nanopore, genomeSize = 3.5 m) (Koren et al. 2017) and Flye v2.9.3 (parameters: --meta) (Kolmogorov et al. 2020). The assembled contigs were then error-polished using racon v1.5.0 with default parameters (Vaser et al. 2017).

Genes were predicted from the metagenome assemblies using Prodigal v2.6.3 (Hyatt et al. 2010). A clustering step was conducted using MMseqs2 v15.6f452 (Steinegger and Söding 2017). The gene catalog was annotated using a modification of the workflow available at https://github.com/lucaz88/FunTraits (Zoccarato et al. 2022). An HMM-based approach using KEGG Orthologs of endoglucanase (K01179) and β-glucosidase (K05349) was employed. The assembly and annotation of the gene catalog were carried out by the BOKU Core Facility Bioinformatics.

### Amplification of cellulase genes by multiplex PCR

The most prominent cellulases from the eDNA (the same samples were used for NGS) were amplified using a multiplex PCR approach. We chose the annealing temperature (T_a_) to be used around the central annealing temperature, and the selection process for the genes to be amplified was also based on the T_a_ being close, with a maximum difference of no more than 2 °C. Using this approach, we chose 16 sets of endoglucanase-specific primers and 10 sets of β-glucosidase-specific primers. Fifty microliters of reaction cocktails contained 0.2 mM dNTPs, 1× reaction buffer (New England Biolabs, Beverly, MA), 1U Q5 polymerase (New England Biolabs) and 150 nM of each primer. The amplicons were then inserted into the pPICZαA vector for *K. phaffii* X33 secretory expression (Invitrogen, Carlsbad, CA) with HiFi DNA assembly (New England Biolabs). NEBUILDER (v. 2.9.1; https://nebuilder.neb.com/#!/, accessed March 2025) was used to design primers. The list of primers used is given in Table S2. Coding sequences were cloned in a way that they contain a C-terminal His6 tag. The products obtained above were purified with the Monarch PCR & DNA Cleanup Kit (New England Biolabs). The GenBank accession numbers of the 16 endoglucanase and 10 β-glucosidase sequences are given in Table S3 in the Supplementary material.

### Phi 29 rolling circle amplification

Obtained recombinant plasmids were transformed directly into *K. phaffii* without an amplification step in *E. coli* (Tachioka et al. 2016). To produce sufficient amounts of DNA for *K. phaffi* genomic integration, recombinant plasmid DNA needs to be amplified by Phi 29 polymerase (New England Biolabs) using RCA (Gibson et al. 2009; Hutchison et al. 2005) facilitated by random primer 6 (New England Biolabs). After 20 h of incubation at 30 °C, the mixture was heated to 65 °C for 10 min to inactivate the polymerase, and DNA amplification was confirmed by electrophoresis in a 0.8% agarose gel. The amplified DNA was then treated with *Blp*I (for endoglucanase) or *Pme*I (for β-glucosidase) (both from New England Biolabs) and a small aliquot was subjected to 0.8% agarose gel electrophoresis. The rest of the digested DNA was precipitated with ethanol for purification. At least 2 µg linearized recombinant plasmid was transformed to *K. phaffi* using a standard electroporation protocol (Invitrogen) in a BioRad MicroPulser Electroporator (BioRad Laboratories, Hercules, CA). The transformed cells were plated on YPD agar with zeocin (100 μg/ml) and incubated at 30 °C for three days.

### 96-well plate cultivation and screening

Single transformant colonies were transferred into deep-well microtiter plates containing 250 µl of YPD media per well and incubated at 30 °C for 3 days with shaking at 350 rpm. Then, 250 µl of YP medium was added. At 70, 82, and 106 h after inoculation, 50 µl of methanol was added for induction of gene transcription via the AOX promoter. At 24 h after the last addition of inducer (for a total cultivation time of 130 h), the cells were harvested by centrifugation at 3500 rpm and 4 °C for 15 min, and the supernatants withdrawn for activity screening.

The supernatants were screened in 96-well plate format (Weis et al. 2004). Endoglucanase activity was determined using carboxymethyl cellulose as substrate and a Dinitrosalicylic Acid (DNS)-based assay (Miller 1959). Cellobiohydrolase activity was measured with 4-Methylumbelliferyl β-D-cellobioside (4-MUC), and 4-Methylumbelliferyl β-D-glucopyranoside (4-MUG) was used for β-glucosidase activity. These assays were adapted to microtiter plates: 80 µl of the provided substrate solution were combined with 20 µl sample (supernatant), mixed, and incubated at 50 °C for 15 min. For the DNS assay, 100 µl DNS was added and the plate was heated in a boiling water bath for 15 min, then the absorbance at 540 nm was measured with a 2300 EnSpire Multilabel Plate Reader (PerkinElmer, Waltham, MA). For the 4-MUC and 4-MUG assays, absorbance was measured with excitation at 360 nm and an emission wavelength of 465 nm. Both assays were offset against a calibration curve generated with known standard concentrations (glucose standard concentrations for the DNS assay and 4-Methylumbelliferyl for the MUG and MUC-based assays).

### Construct confirmation

Colonies presenting cellulase activity were confirmed by colony PCR. A single colony of each positive strain was used as DNA template for a 50 µl PCR reaction, containing 0.2 mM dNTPs, 1× reaction buffer, 1 U Q5 polymerase (all from New England Biolabs), 500 nM each of the pPICZαA universal forward primer (5′ TTAGCTTACTTTCATAATTGC 3′) and pPICZαA universal reverse primer (5′ AATGAAGCCTGCATCTCTCAG 3′). The following program was used for PCR: 5 min at 98 °C, 35 cycles with 10 s at 98 °C, 30 s at 56 °C and 1.5 min at 72 °C, followed by a final extension step of 2 min at 72 °C. After gel electrophoresis, the identity of the resulting PCR products was verified by DNA Sanger sequencing.

### SDS PAGE and Western Blot

Representatives of each colony with different active cellulases that were confirmed by sequencing were selected, the supernatants concentrated using Amicon Ultra 0.5 Centrifugal Filters (Sigma Aldrich, St. Louis, MO) and analyzed by SDS PAGE for endoglucanase and β-glucosidase using Mini-PROTEAN® TGX™ Precast Gels (BioRad Laboratories, Hercules, CA). Forty micrograms of protein was loaded per lane. For β-glucosidase, we also performed western blot using Penta-His Tag Monoclonal Antibody (Invitrogen, Carlsbad, CA) as primary antibody, and Polyclonal Rabbit Anti Mouse Immunoglobulin/HRP (Agilent Technologies, Santa Clara, CA) as secondary antibody. SDS PAGE units and blotting machine were from BioRad, visualization was done on a Gel Doc XR + Gel Documentation System (BioRad Laboratories, Hercules, CA).

### Sequence analysis

All cellulase genes identified through Sanger sequencing were analyzed for sequence similarity using BLAST search against the non-redundant (nr) GenBank, Uniprot/Swissprot, and Protein Data Base from NCBI. A phylogenetic tree was inferred using the Maximum Likelihood method implemented in the program MEGA12.1 (Kumar et al. 2024).

## Results

### Amplification of cellulase genes by multiplex PCR and RCA

**Fig. 1:**
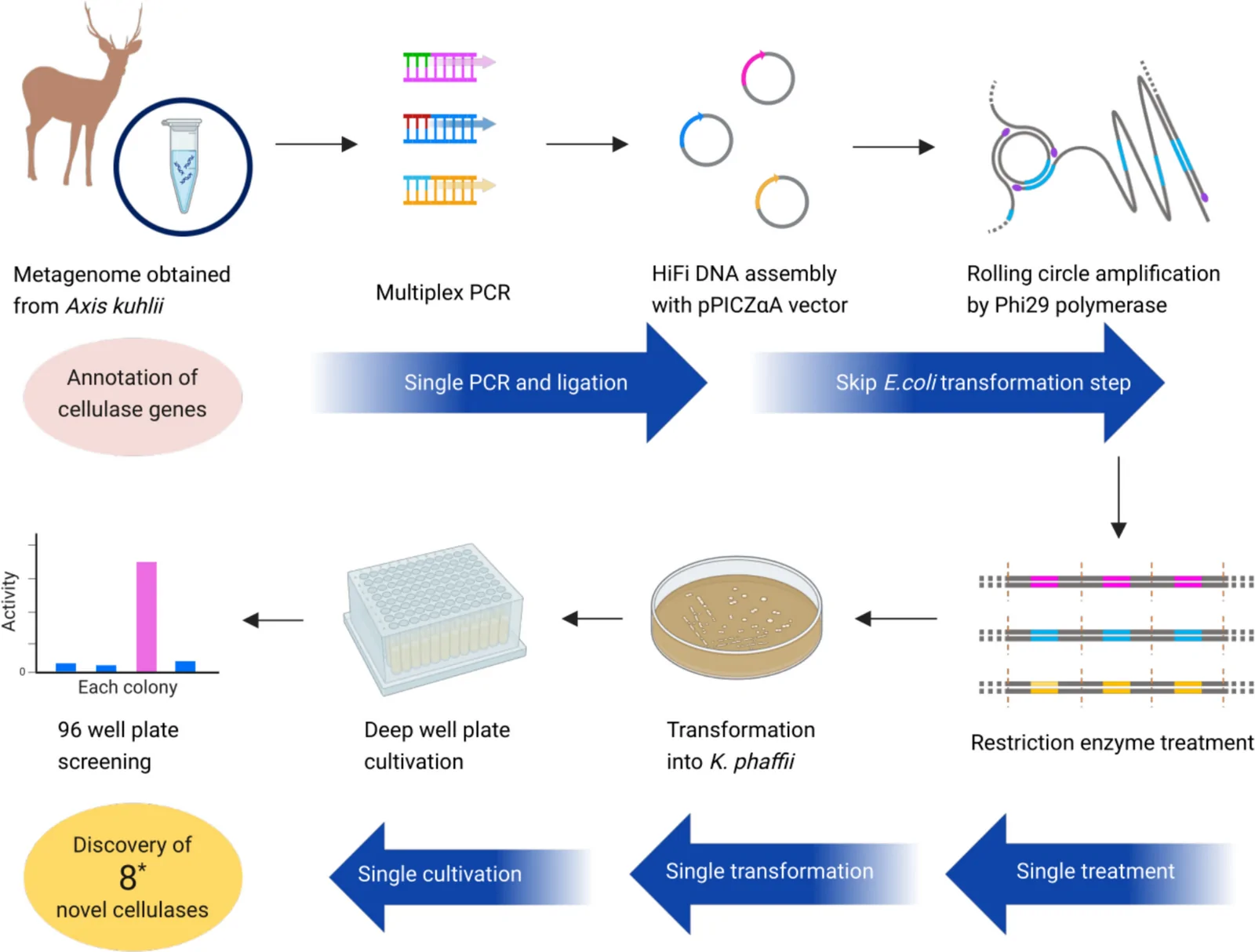
Scheme of metagenomic strategies for the identification of novel cellulases from environmental samples. Figure created with Biorender.com and SILHOUETTE DESIGN. *We carried out this scheme for endoglucanases and β-glucosidases and found 8 novel cellulases in total

**Fig. 2:**
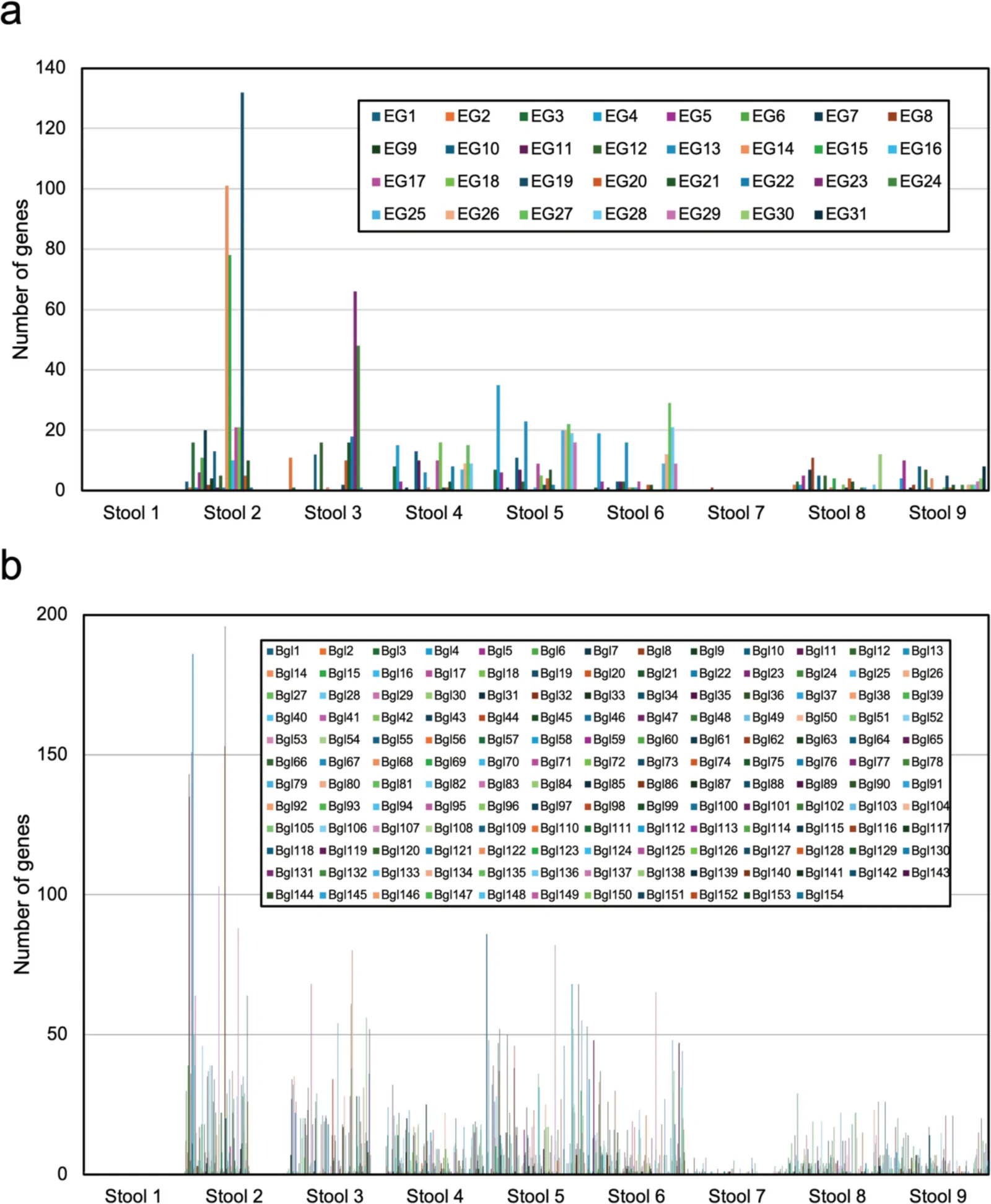
Abundance of cellulases from 9 stool samples of *A. kuhlii*. **a** Endoglucanase and **b** β-glucosidase

We performed NGS on eDNA samples, then screened for and identified cellulase-encoding genes (Fig. 1). Metagenome data were obtained from nine stool samples of *Axis kuhlii* collected from the Nature Reserve and Wildlife Reserve on Bawean Island, Gresik, East Java, Indonesia; 31 endoglucanase and 154 β-glucosidase-encoding genes with different abundance distributions were identified in each sample (Fig. 2). We used the DNA from stool sample 5 for endoglucanase screening and stool sample 2 for β-glucosidase screening, because these samples had the highest cellulase distribution and abundance compared to the others.

For endoglucanases, from the 31 genes found in the sample, we selected genes with 5′- and 3′-termini allowing the design of oligonucleotide primers that had an annealing temperature in the range of 62–64 °C for multiplex PCR purposes, leaving 16 genes. For β-glucosidase, from the 154 genes found in the sample, we selected the ten most abundant genes and those representing different annotations in the sample, which had an annealing temperature for the multiplex primers of 64–66 °C (Table S1).

Multiplex PCR resulted in bands approximately 970–1000 bp in size with endoglucanase-specific primers, and approximately 2200–2300 bp in size with β-glucosidase-specific primers. The amplicons were purified and ligated with linearized pPICZαA with HIFI DNA Assembly to be used as plasmid template for rolling circle amplification. After cutting with *Blp*I (for endoglucanase) and *Pme*I (for β-glucosidase), the DNA fragments from the RCA, which were initially larger than 10,000 bp (outside the separation range of the agarose gel), resulted in dominant DNA fragments of approximately 4400 bp for endoglucanase and approximately 5600 bp for β-glucosidase (Fig. S1). These sizes correspond to the pPICZalphaA backbone (3400 bp) plus endoglucanase- or β-glucosidase-encoding genes.

### Cultivation and screening for active enzymes in *K. phaffii* using 96-well plate format

**Fig. 3:**
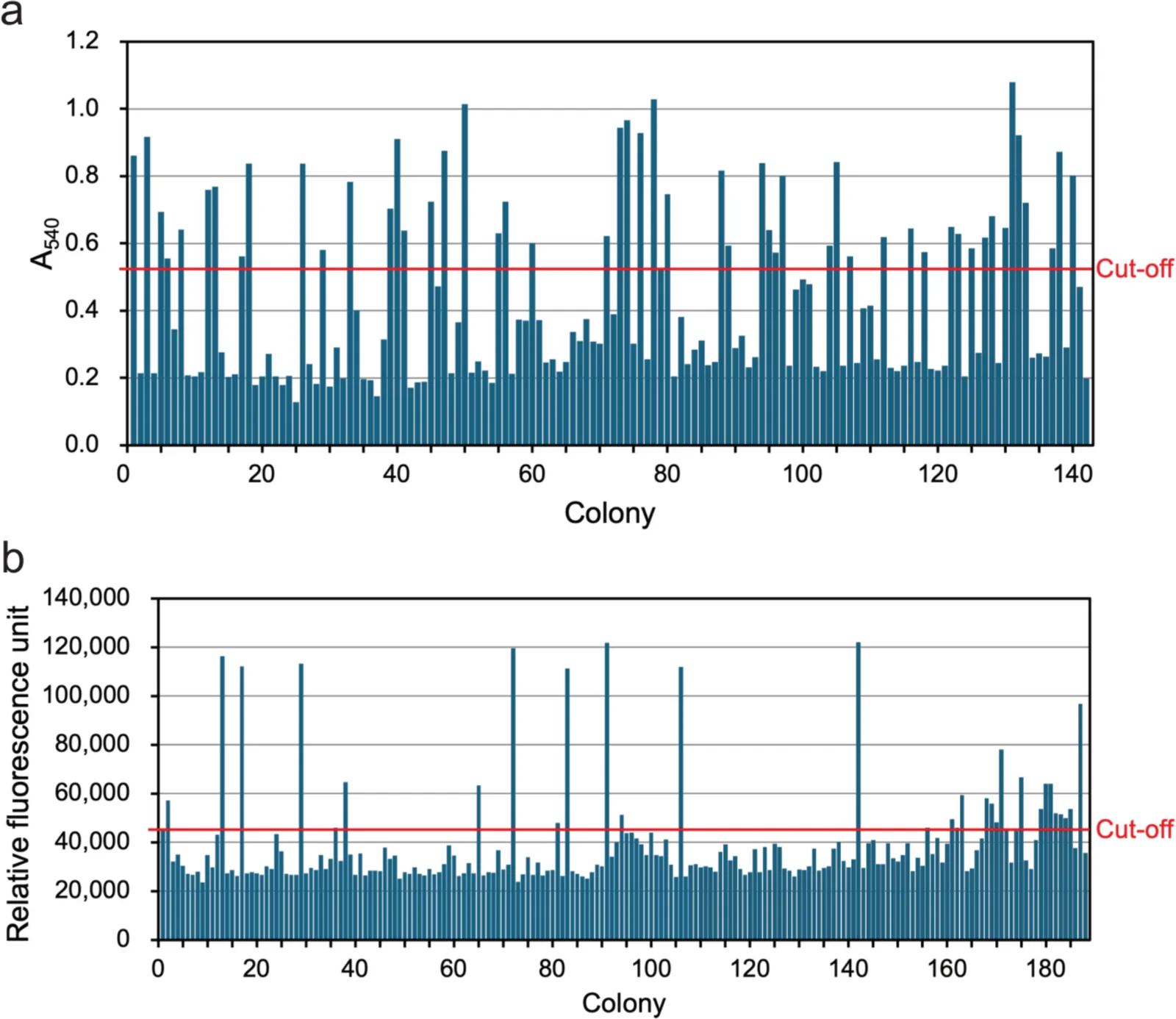
Screening cellulases. **a** CMC assay for endoglucanase and **b** 4MUG assay for β-glucosidase. The last bar indicates the negative control

After successful transformation into *K. phaffii*, more than 150 transformants for both endoglucanase- and β-glucosidase-encoding genes were cultivated in 96 plates for cellulase activity screening. Colonies with highest activity above the threshold were selected for further testing and sequencing, namely 32 colonies containing an endoglucanase gene and 24 colonies containing a β-glucosidase gene (Fig. 3). The metagenome-derived inserts were identified by Sanger sequencing. 31 (out of 32) colonies harbored endoglucanase genes, and 11 (out of 24) harbored β-glucosidase genes.

Endoglucanase genes show a reasonably good ratio when comparing gene richness in the metagenomic samples to functional genes as transformants in *K. phaffii*. The number of DNA copies in the DNA template directly correlates with the number of transformants for each endoglucanase gene. There were 35 copies of the endoglucanase EG Q4 gene in the metagenome library, which produced 12 colonies of transformants with this gene. Similar to EG Q25, which had 20 gene copies in the DNA template, EG13 produced five colonies from 23 copies in the library. While the single transformant colony from seven gene copies of EG Q11 in the metagenomic template is below the otherwise observed ratio, eight transformant colonies for endoglucanase EG Q28 are somewhat numerous for the 19 templates. We also find that the number of copies in the metagenome template and the number of transformant colonies that contain the corresponding β-glucosidase genes are nearly equal. The ratio is significantly lower than that of the endoglucanase genes, though, as only eight colonies containing BGL1 were found out of the original 196 gene copies in the metagenome template. Only two colonies were obtained for BGL2, despite the abundance of 186 gene copies, whereas one transformant clone for BGL25 was produced from 30 gene copies, which is a roughly similar ratio (Table 1).

**Table 1:** Diversity of cellulase genes in metagenome samples and transformants

	Metagenome sample		Transformants	
Endoglucanase	Number of genes	Ratio (%)	Number of colonies	Ratio (%)
EG4	35	34	12	39
EG11	7	7	1	3
EG13	23	22	5	16
EG25	20	19	5	16
EG28	19	18	8	26
Total	104	100	31	100
β-Glucosidase				
BGL 1	196	48	8	73
BGL 2	186	45	2	18
BGL 25	30	7	1	9
Total	412	100	11	100

### SDS PAGE of five endoglucanases and three β-glucosidases

**Fig. 4:**
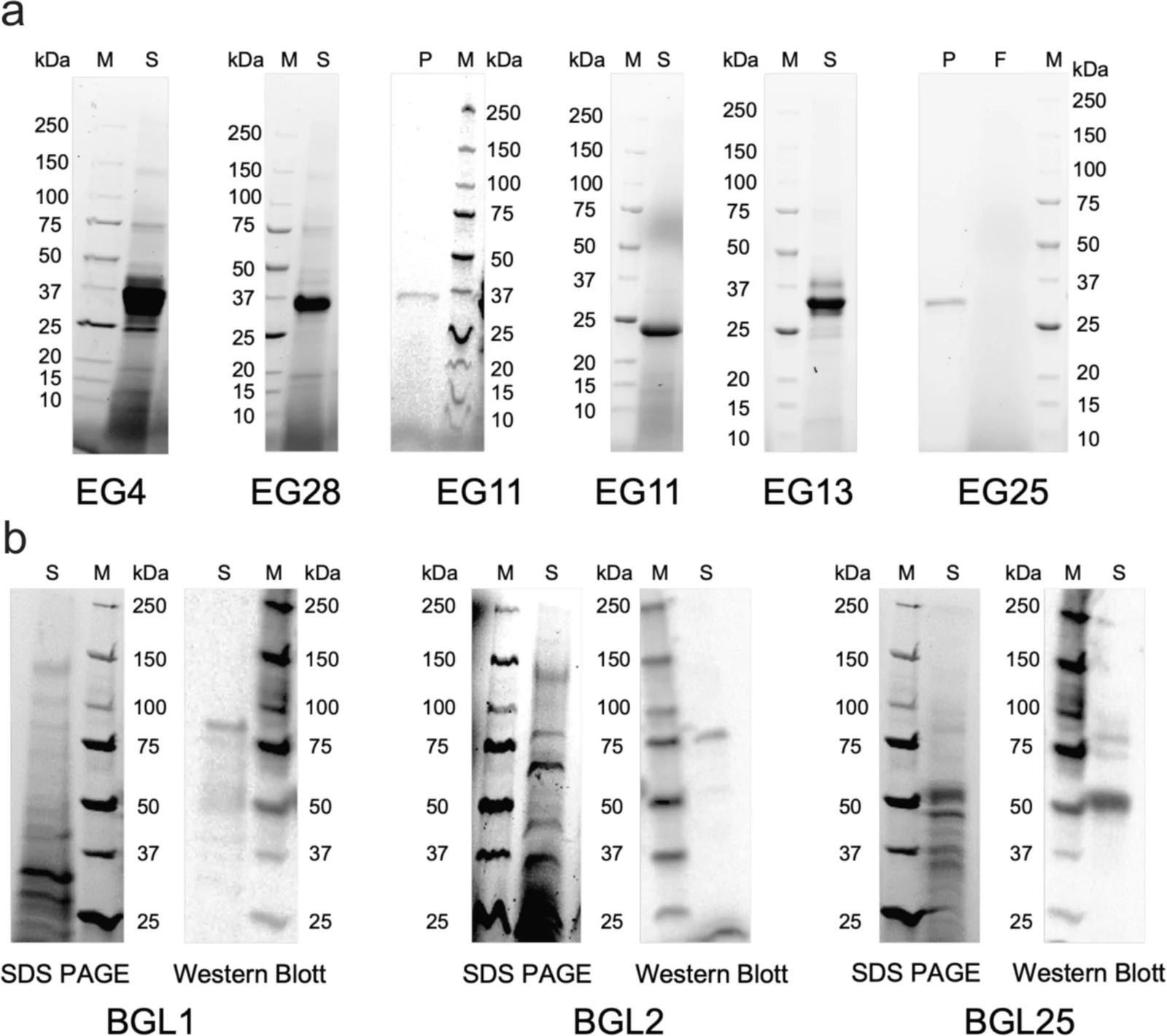
**a** SDS PAGE for endoglucanase and **b** SDS PAGE and Western blot for β-glucosidase. M, marker; S, supernatant; P, pure; F, flow-through

The SDS PAGE results for endoglucanases show clear and dominant bands with a size ~ 34 kDa without prominent non-specific proteins, indicating optimal production of this protein in *K. phaffi*. Only in EG 11 the SDS PAGE showed a band indicating a smaller size (24 kDa instead of 34 kDa) in some, but not all cases. Expression of β-glucosidase genes was not as strong as that of endoglucanase genes, so the bands on SDS PAGE are not clearly visible. Therefore, Western blotting was performed to confirm the identity of these proteins (~83 kDa) (Fig. 4).

### Rapid screening of colonies for other properties using a 96-well plate

**Fig. 5:**
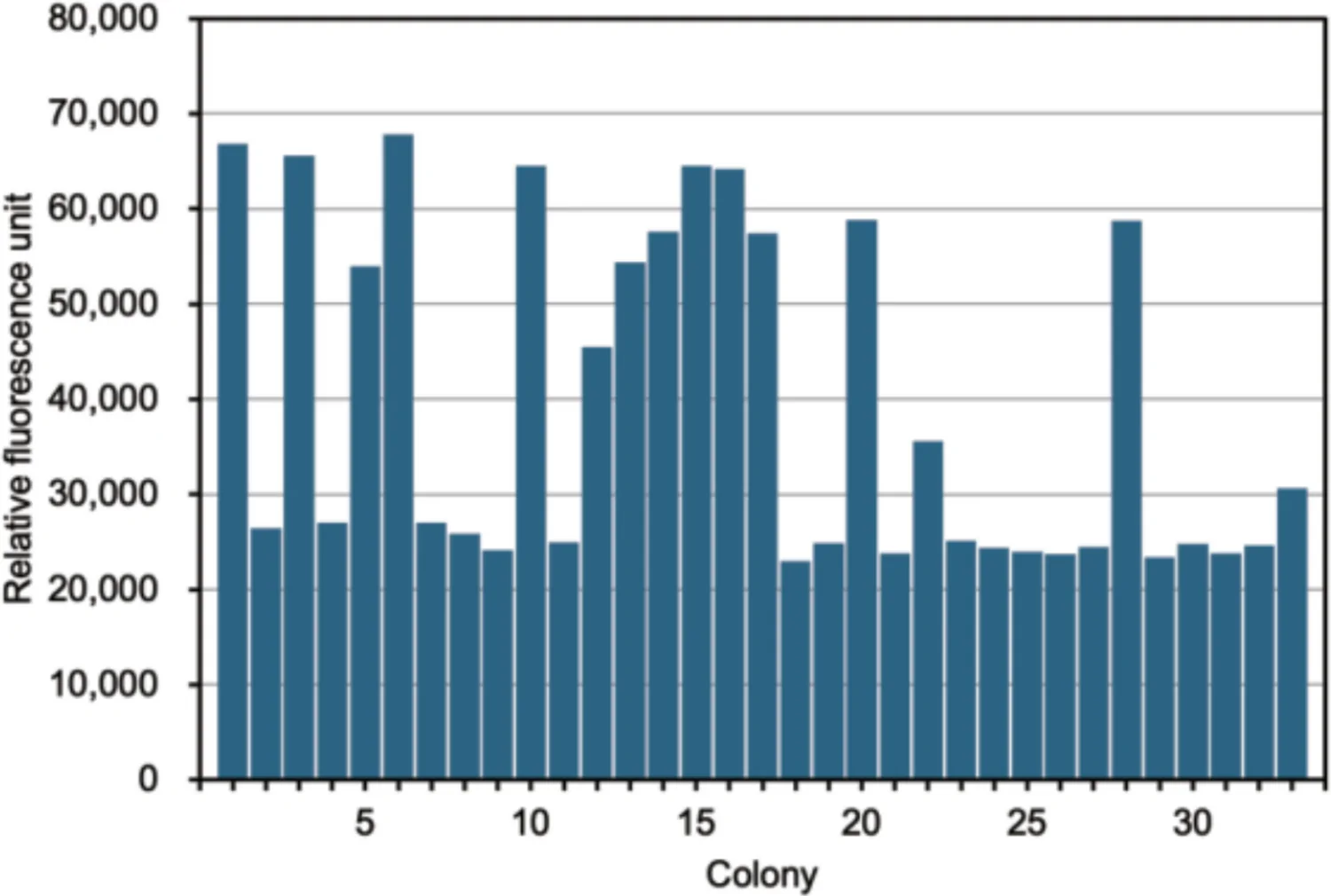
Screening exoglucanase (cellobiohydrolase) activity using 4MUC in endoglucanase transformants. The last bar indicates the negative control

We used a 96-deep well plate format to screen the obtained clones for activity with other substrates. Thirty-two colonies with high endoglucanase activity were screened for exoglucanase activity using 4MUC as a substrate (Fig. 5). Thirteen clones were found to have exoglucanase activity, and all these contained endoglucanase genes 4, 13, 25, and 28. We also found one endoglucanase colony that could hydrolyse xylan (Fig. S2a), indicating that the endoglucanase it contained had promiscuous xylanase activity. Additionally, we could identify several colonies showing higher activity not only at standard pH and temperature conditions, but also at acidic and alkaline pH (pH 5.0 and 8.0, respectively) (Fig. S2b), as well as at elevated temperatures (60 °C) (Fig. S2c).

### BLAST sequence comparisons

**Fig. 6:**
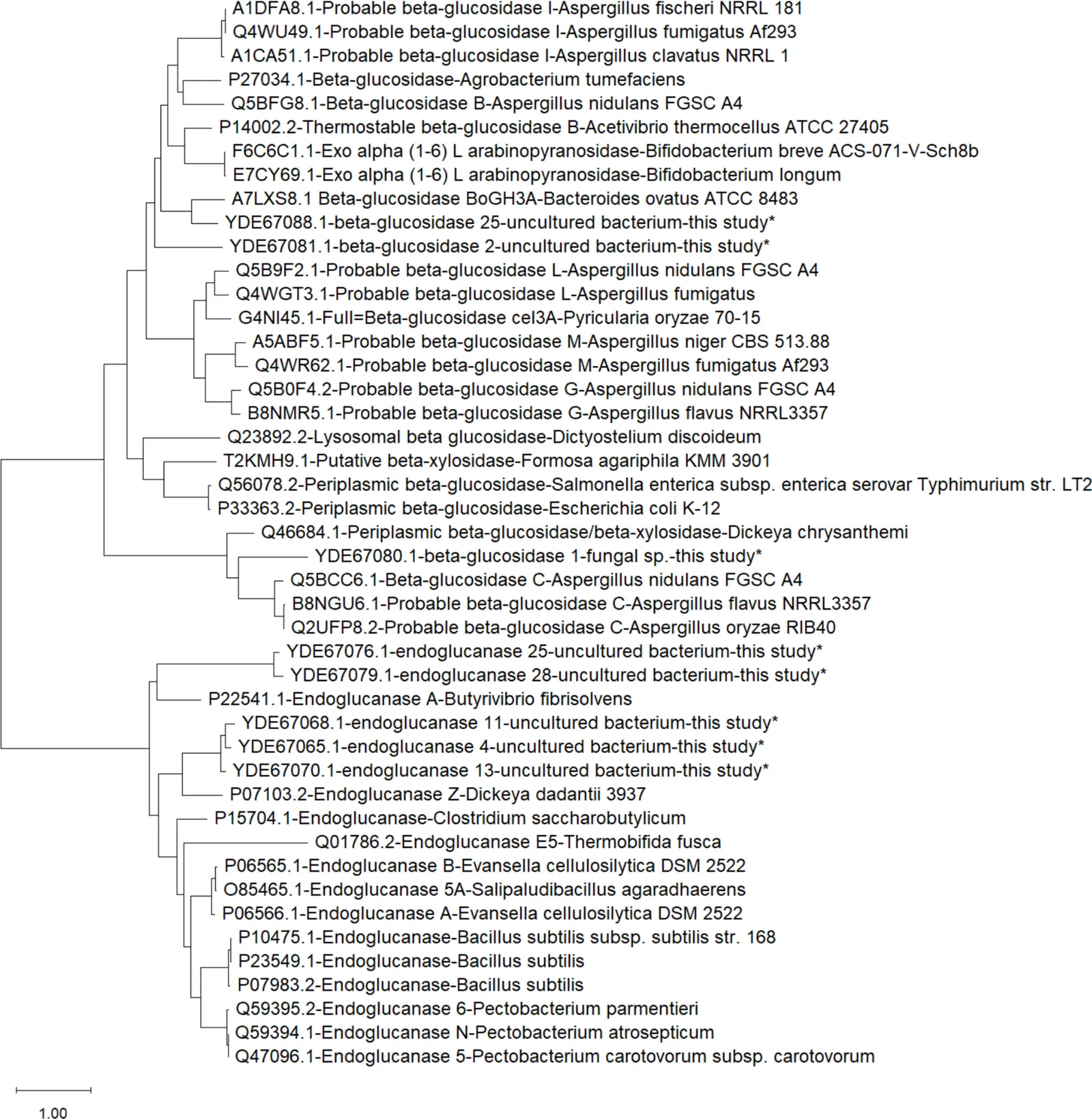
Phylogenetic tree inferred from the eight sequences resulting in active enzymes and the ten best BLAST hits for each one. The sequences described in this study are marked with an asterisk

From the BLAST results with three different databases (non-redundant GenBank, Uniprot-Swissprot, and PDB), nr GenBank has the highest sequence query coverage and identity percentages because this database includes all sequences submitted to the database (such as NGS sequences, metagenome sequencing projects). In order to assess the novelty of the identified cellulase encoding genes, Uniprot-Swissprot and PDB are more appropriate (Table 2). The five endoglucanase genes that were successfully expressed showed similarity with sequences from the UniProtKB/SwissProt database below 50% (24% to 45%) and with sequences from the PDB database below 52% (between 41 and 51%). For β-glucosidase, the three enzymes we obtained showed sequence similarity with sequences from the UniProtKB/SwissProt database between 32 and 51%, and with the PDB database between 33 and 52%. From the eight sequences producing active enzymes and the ten best BLAST hits for each one (corrected for redundancy), a phylogenetic tree was inferred (Fig. 6).

**Table 2:** Blast results with nr GenBank, Uniprot-Swissprot, and PDB databases

No.	Query	Database	Description	Organism	Cov (%)	ID (%)	Accession
**Endoglucanase**							
		GenBank	CAZy GH 5	*Bacteroidales bacterium*	99	88	MBR2064430.1
1	EG Q4	UniProtKB-SwossProt	Endoglucanase Z, Cellulase Z	*Dickeya dadantii*	87	45	P07103.2
		PDB	Chain A, Endoglucanase	*Cytophaga hutchinsonii*	100	42	5IHS_A
		GenBank	CAZy GH 5	*Bacteroidales bacterium*	96	98	MBE6235115.1
2	EG Q13	UniProtKB-SwossProt	Endoglucanase Z, Cellulase Z	*Dickeya dadantii*	92	43	P07103.2
		PDB	Endoglucanase	*Cellvibrio japonicus*	92	45	8BQA_AAA
		GenBank	CAZy GH 5	*Bacteroidales bacterium*	99	100	MBE6235412.1
3	EG Q25	UniProtKB-SwossProt	Endoglucanase Z, Cellulase Z	*Butyrivibrio fibrisolvens*	72	24	P22541.1
		PDB	Cellulase, putative, cel5C	*Cellvibrio japonicus*	91	51	8BN7_AAA
		GenBank	Cellulase family GH	*Bacteroidales bacterium*	99	88	MBO5016517.1
4	EG Q28	UniProtKB-SwossProt	Endoglucanase A, Cellulase A	*Butyrivibrio fibrisolvens*	62	26	P22541.1
		PDB	Cellulase, putative, cel5C	*Cellvibrio japonicus*	91	49	8BN7_AAA
		GenBank	CAZy GH 5	*Bacteroidales bacterium*	99	84	MEE0236234.1
5	EG Q11	UniProtKB-SwossProt	Endoglucanase Z, Cellulase Z	*Dickeya dadantii*	92	42	P07103.2
		PDB	Endoglucanase, CAZy GH 5	*Cytophaga hutchinsonii*	100	41	5IHS_A
**β-Glucosidase (BGL)**							
		GenBank	CAZy GH 3	*Bacteroidales bacterium*	100	99	MBQ3251508.1
1	BGL 1	UniProtKB-SwossProt	Probble β-glucosidase C	*Aspergillus flavus*	90	32	B8NGU6.1
		PDB	β-Glucosidse	*Clavibacter michiganensis*	92	33	9UL7_A
		GenBank	CAZy GH 3	*Bacteroidales bacterium*	100	99	MBQ8810697.1
2	BGL 2	UniProtKB-SwossProt	Thermostble β-glucosidase B	*Acetivibrio thermocellus*	92	37	P14002.2
		PDB	β-Glucosidase	*Segatella copri*	93	52	8IDZ_A
		GenBank	CAZy GH 3	*Bacteroidales bacterium*	100	100	MBO5108178.1
3	BGL 25	UniProtKB-SwossProt	β-Glucosidse CAZy GH 3	*Bacteroides ovatus* ATCC 8483	100	51	A7LXS8.1
		PDB	Thermostble β-glucosidase B	*Acetivibrio thermocellus* AD2	95	41	7MS2_A

## Discussion

In this study, we successfully identified eight novel cellulases, five endoglucanases, and three β-glucosidases from the metagenome of the highly threatened Indonesian deer *Axis kuhlii*. By combining multiplex PCR of genes annotated as cellulases with RCA and functional screening in *K. phaffii*, functional enzymes were identified within 2 weeks after gene annotation. Conventional reference-based metagenomic screening approaches typically involve selecting a number of candidate genes and constructing individual expression strains one by one (Oliva et al. 2024; Paloyan et al. 2025). This workflow substantially reduced the required time and labor. The rapid identification of multiple functional cellulases highlights the efficiency of this strategy for enzyme discovery from eDNA.

The discovery of several cellulases using our method demonstrates that multiplex PCR using eDNA as a template can work successfully and can be a cost-saving alternative compared to approaches using custom-synthesized genes. For successful multiplex PCR, primer-annealing temperature (T_a_) interactions can impact multiplex performance (Lafrance et al. 2023). We designed primers with a maximum difference in T_a_ of no more than 2 °C and set the T_a_ for the annealing step around the central T_a_ for all primers. In addition to T_a_, substantial differences in target sequence length may result in amplification bias, since longer templates require extended elongation times and are amplified less efficiently. A typical limitation of reference-based metagenomics is the difficulty to discover novel enzymes, which must rely on selecting target genes with relatively low similarity to known sequences. For example, an oxidative enzyme cleaving cellulose, representing a previously unknown class compared with known enzymes, was found in the metagenome of long-term sugarcane bagasse-covered soil in Brazil by screening for candidate genes with a similarity of at least 10% identity with known carbohydrate-processing proteins (Santos et al. 2025). Although targeting genes with low sequence homology carries a higher risk of failure, multiplex PCR enables the simultaneous evaluation of both high- and low-risk candidates at once. Changing the portfolio of included target genes in multiplex PCR allows to balance between impact and likelihood of success. This strategy reduces experimental bias toward well-characterized enzymes and increases the likelihood of identifying novel functions.

*Komagatella phaffii* is a eukaryotic expression system that is capable of post-translational modifications and has a powerful secretory system (Vijayakumar and Venkataraman 2023). However, transformation of *K. phaffii* generally requires several micrograms of plasmid DNA, which is substantially higher than that required for *E. coli*. This is generally done by transformation of *E. coli* after plasmid assembly, followed by cultivation and plasmid preparation. For the endoglucanase genes presented here, the conventional workflow requires 16 transformations into *E. coli* and *K. phaffii* individually. We applied rolling circle amplification (RCA) to generate sufficient amounts of plasmid DNA and transformed the reaction products digested by the restriction enzyme into *K. phaffi* at once. Successful RCA requires a completely ligated circular plasmid. Accordingly, ligation-based cloning methods, including HiFi DNA Assembly and Golden Gate Assembly, are compatible with RCA (Fryer et al. 2024) , whereas seamless cloning systems that do not involve ligation, such as In-Fusion™ assembly (Zhu et al. 2007), are unsuitable. Overall, our approach can considerably shorten the process of expressing multiple genes derived from metagenomic samples in *K. phaffii*.

Functional screening was successful since the *K. phaffii* transformants contained various genes. The ratio of the transformants approximately reflected the numbers of the template gene copies in the metagenome sample in case of both endoglucanases and β-glucosidases. This suggests that multiplex PCR and RCA amplified different template genes almost equally for at least the positive target genes. Thus, the dominant gene in a template tends to be dominant in the population of transformants, although the ratio of all genes is effective for screening all target genes. Optimization of the primer concentration ratio (Lafrance et al. 2023) could be a solution to control the ratio of transformant genes. It should be noted that 180 transformants containing β-glucosidase genes proliferated, the majority of which might encode β-glucosidases with no activity (7 out of 10 amplified genes). Only three genes (BGL 1, 2, and 25) were represented by eleven colonies containing active β-glucosidase. This also holds true for endoglucanase, where only five of sixteen endoglucanase genes produced clones with enzymatic activity. The reason for negative results can be a lack of amplification of the template genes, a failure of recombinant protein production or an inactive protein. The amplification process may favor genes that amplify easily, whereas genes with unfavourable CG-content or other problematic characteristics will produce fewer amplicons, which will be reflected in the quantity of intact expression plasmids. The generation of mini libraries through multiplex PCR amplification from eDNA as a template does not involve codon optimization (as custom gene synthesis would). This can result in a lower concentration of active enzyme in the supernatant, even if mRNA levels are comparable (Hanson and Coller 2018). Under unfavourable conditions, this may result in an assay readout that is near or below the detection limit, causing candidate genes to be missed. The complexity of using metagenomic DNA as a source of producing protein also can lead to difficulties in transcription and translation within hosts, often resulting in insufficient expression of foreign genes (Uchiyama and Miyazaki 2009). Even though we were able to obtain multiple functional enzymes using this method, this can be seen as a limitation.

The screening format in 96-well microtiter plates, which allows the processing of a greater number of constructs and screens for multiple properties (different substrates, optimal pH) in a standardized process, is another benefit of this strategy (Shang et al. 2018). For colonies transformed with endoglucanase genes, activity screening was performed not only using the DNS assay with CMC as a substrate, but also for cellobiohydrolase and xylanase activity, and optimal pH and thermostability screening could be performed as well. The majority of the active cellulase enzymes that we were able to obtain do not exhibit significant similarity to sequences found in the PDB and UniProtKB/SwissProt databases. BLAST results with UniProtKB/SwissProt and PDB show that our endoglucanases show a similarity with characterized enzymes between 24 and 51%. Similar functional metagenomic studies have obtained endoglucanase and xylanase genes from soil with 39% similarity (Chai et al. 2020), from Chinese yak rumen with 72% similarity (Chang et al. 2011), and from goat rumen with 56–59% similarity (Thapa et al. 2023). Our β-glucosidase samples showed similarities between 32 and 52% compared to 46–57% from kusaya gravy (Uchiyama et al. 2015), 87% from soil (Lu et al. 2013), and 52% from compost metagenome (Uchiyama et al. 2013). The BLAST results further indicate that most cellulases are bacterial enzymes. One β-glucosidase gene (BGL1) is more similar to eukaryotic sequences (β-glucosidase C from *Aspergillus flavus*). *K. phaffii* is generally suited for eukaryotic genes, especially genes encoding secretory proteins that require post-translational modifications, but it can also express many prokaryotic genes (Lv and Cai 2025). These and our results demonstrate that the use of *K. phaffi* can lead to successful expression of metagenomic genes and yield functional enzymes even when the metagenome samples are assumed to be dominated by prokaryotic DNA.

*Komagatella phaffii* is a well-established host platform for the secretory production of recombinant proteins (Weis et al. 2004). If a gene found in this way satisfies specific requirements, positive clones could be transferred to process optimization and upscaling quickly. With a few minor modifications, like the recircularization procedure prior to transformation, the workflow can be transferred to other host systems, such as *Bacillus subtilis*-based platforms. In order to maintain the expression construct as a circular, autonomously replicating plasmid rather than integrated into the genome as is required for *K. phaffii*, a recirculation with ligase is necessary because the DNA fragment obtained prior to transformation is a linear fragment. The same sets of substrate analogues for fluorescent detection can be used for secretory enzymes; for non-secretory enzymes an adaptation to *E. coli* is feasible as well, if an appropriate screening assay is available.

## Conclusion

With the approach presented in this study, which combines metagenomics sequencing, multiplex PCR, rolling circle amplification, and the use of semi-high-throughput assays with deep well plates for rapid screening, we were able to identify novel variants of two different enzyme families encoded in environmental samples more quickly and cost-efficiently. With samples sourced from the environment, this approach covers all genes represented in microorganisms, including those that cannot be cultivated. This approach has proven effective for two different enzyme groups, namely endoglucanase and β-glucosidase, and can be applied for other enzyme groups as well.

## Supplementary Information

Below is the link to the electronic supplementary material.

ESM 1 PDF (454 KB)

## Data Availability

The data are given in the text and the supplemented files.
