# Supplementary material for: A fast workflow to explore active enzymes from environmental samples through functional metagenomics
Source: Appl Microbiol Biotechnol. 2026 Mar 25;110(1):125. doi: 10.1007/s00253-026-13805-1 (PMC13021860; doi:10.1007/s00253-026-13805-1)
Supplement: Supplementary file 1 — PDF (454 KB) [file 253_2026_13805_MOESM1_ESM.pdf]

Supplementary Material

Muammar et al., A Fast Workflow to Explore Active Enzymes from Environmental Samples Through Functional Metagenomics

Contents:

Table S1 Cellulase Genes Selection for Multiplex PCR

Table S2 List of primers of Endoglucanase and β-glucosidase for HIFI Assembly to pPICZαA

Table S3 List of GenBank accession numbers of the expressed sequences

Fig. S1 Electrophoresis Results of Amplified Cellulases by Multiplex PCR

Fig. S2 Screening of colonies

Fig. A1 Original uncropped pictures of SDS PAGE of endoglucanase samples

Fig. A2 Original uncropped pictures of SDS PAGE of β-glucosidase samples

Table S1 Cellulase Genes Selection for Multiplex PCR

A. Selected Endoglucanases (Annealing Temperature between 62-64 C) in Stool Sample 5

**Query**	**Gene Length**	**Stool sample number:**									**Ann T**	**KEGG Ortholog definition**	**Selected**
		**1**	**2**	**3**	**4**	**5**	**6**	**7**	**8**	**9**			
**EG1**	1089	0	3	0	0	0	0	0	0	0	53	hypothetical protein	X
**EG2**	1008	0	1	11	0	0	0	0	2	0	63	glycoside hydrolase family 5	X
**EG3**	1809	0	16	1	8	7	1	0	3	0	63	polysaccharide deacetylase	V
**EG4**	972	0	1	0	15	35	19	0	2	4	63	eglS; Endoglucanase precursor	V
**EG5**	1137	0	6	0	3	6	3	0	5	10	63	Endoglucanase C307 precursor	V
**EG6**	1608	0	11	0	0	0	0	0	0	0	56	celA_1; Endoglucanase A precursor	X
**EG7**	1008	0	20	0	1	1	1	0	7	1	64	glycoside hydrolase family 5	V
**EG8**	1005	0	2	0	0	0	0	1	11	2	56	glycoside hydrolase family 5	X
**EG9**	1113	0	4	0	0	0	0	0	0	0	61	dihydroxy-acid dehydratase	X
**EG10**	972	0	13	12	13	11	3	0	5	8	69	eglS; Endoglucanase precursor	X
**EG11**	978	0	1	0	10	7	3	0	0	0	63	eglS; Endoglucanase precursor	V
**EG12**	1233	0	5	16	0	3	3	0	5	7	64	Endoglucanase C307 precursor	V
**EG13**	975	0	1	0	6	23	16	0	0	1	63	eglS; Endoglucanase precursor	V
**EG14**	972	0	101	1	1	0	1	0	1	4	72	eglS; Endoglucanase precursor	X
**EG15**	996	0	78	0	0	0	1	0	4	0	72	glycoside hydrolase family 5	X
**EG16**	948	0	10	0	0	1	1	0	0	0	63	eglS; Endoglucanase precursor	V
**EG17**	1242	0	21	0	10	9	3	0	0	0	70	Endoglucanase C307 precursor	X
**EG18**	954	0	21	0	16	5	0	0	2	1	63	eglS; Endoglucanase precursor	V
**EG19**	1416	0	132	2	1	2	0	0	1	5	63	Endoglucanase C307 precursor	V
**EG20**	1011	0	5	10	1	4	2	0	4	1	70	glycoside hydrolase family 5	X
**EG21**	939	0	10	16	3	7	2	0	3	2	64	eglS; Endoglucanase precursor	V
**EG22**	975	0	1	18	8	2	0	0	0	0	62	eglS; Endoglucanase precursor	V
**EG23**	1257	0	0	66	0	0	0	0	0	0	64	Endoglucanase C307 precursor	X
**EG24**	1254	0	0	48	0	0	0	0	1	2	71	glycoside hydrolase family 5 protein	X
**EG25**	1005	0	0	1	7	20	9	0	1	0	63	glycoside hydrolase family 5	V
**EG26**	1194	0	0	0	9	20	12	0	0	2	63	hypothetical protein	V
**EG27**	1218	0	0	0	15	22	29	0	0	2	64	glycoside hydrolase family 5 protein	V
**EG28**	1008	0	0	0	9	19	21	0	2	2	63	glycoside hydrolase family 5	V
**EG29**	1839	0	0	0	0	16	9	0	0	3	58	polysaccharide deacetylase	X
**EG30**	1239	0	0	0	0	0	0	0	12	4	65	glycoside hydrolase family 5 protein	X
**EG31**	1089	0	0	0	0	0	0	0	0	8	60	hypothetical protein	X

B. Selected β-glucosidase (Annealing Temperature between 64-66 C) in Stool Sample 2

**Query**	**Gene Length**	Stool sample number: **1**	**2**	**3**	**4**	**5**	**6**	**7**	**8**	**9**	**AnnT**	**KO definition**	**Selected**
**b1**	2319	0	196	21	25	24	16	1	12	17	65	beta-glucosidase-like glycosyl hydrolase	V
**b2**	2340	0	186	32	21	26	14	1	7	8	64	beta-glucosidase	V
**b3**	2238	0	153	1	0	1	2	0	0	2	66	glycoside hydrolase family 3 domain protein	V
**b4**	2283	0	151	34	32	39	48	6	14	17	68	glycoside hydrolase family 3 protein	X
**b5**	2238	0	143	0	0	0	0	0	0	0	65	glycosyl hydrolase	V
**b6**	2322	0	135	0	1	2	0	0	1	0	65	bglX_7; Periplasmic beta-glucosidase precursor	V
**b7**	2442	0	103	0	1	0	0	0	0	0	65	beta-glucosidase	V
**b8**	2400	0	88	5	9	7	13	0	3	5	64	bgl3E; beta-glucosidase Bgl3E	V
**b9**	2400	0	64	26	1	1	1	0	0	2	69	beta-glucosidase	X
**b10**	2433	0	64	0	4	1	3	0	1	0	69	beta-glucosidase	X
**b11**	2262	0	50	0	14	10	8	2	29	20	66	bglX; beta-glucosidase BglX	V
**b12**	2337	0	46	20	13	7	10	1	4	2	69	beta-glucosidase-like glycosyl hydrolase	X
**b13**	2262	0	39	2	0	0	1	0	11	2	69	hypothetical protein	X
**b21**	2913	0	35	0	2	1	0	0	1	1	65	Xylan 1,4-beta-xylosidase	X
**b25**	2238	0	30	0	14	8	7	0	4	2	66	bglB_1; Thermostable beta-glucosidase B	V
**b35**	2592	0	22	5	4	4	1	0	4	3	64	bglX_4; Periplasmic beta-glucosidase precursor	V

Table S2 List of primers of Endoglucanase ad β-glucosidase for HIFI Assembly to pPICZαA

No	Primer Name	Sequence (5'-->3')	AnnT (C)	Gene
1	Lin pPICZaA_fwd	catcatcatcatcatcattgagtttg	58	pPICZaA Linearization
2	Lin pPICZaA_rev	tcttttctcgagagatacc	58	pPICZaA Linearization
3	Q3_PD_fwd	gggtatctctcgagaaaagaggacaatggataaggattaacca	63	endoglucanase
4	Q3_PD_rev	caatgatgatgatgatgatgtttattgtttttagcctgttttgcgc	63	endoglucanase
5	Q4_EG_fwd	gggtatctctcgagaaaagatgcgcgaagcaggttc	63.1	endoglucanase
6	Q4_EG_rev	caatgatgatgatgatgatgtaactgatcttttacatacttgccc	63.1	endoglucanase
7	Q5_EG_fwd	gggtatctctcgagaaaagaattacttcgttcagttacgtattgc	62.6	endoglucanase
8	Q5_EG_rev	caatgatgatgatgatgatgtttcatgcgaagggatctg	62.6	endoglucanase
9	Q7_GH5_fwd	gggtatctctcgagaaaagatcctcaaatcctgatcaggac	64,2	endoglucanase
10	Q7_GH5_rev	caatgatgatgatgatgatgttcaacagcttttctttgaagatagc	64,2	endoglucanase
11	Q11_EG_fwd	gggtatctctcgagaaaagatgcagcccgaaatctc	63	endoglucanase
12	Q11_EG_rev	caatgatgatgatgatgatgtaagttattctttacatatttaccccaaggtc	63	endoglucanase
13	Q12_EG_fwd	gggtatctctcgagaaaagatgcactcagaagccgcaa	63,8	endoglucanase
14	Q12_EG_rev	caatgatgatgatgatgatgtttcatgcggagggattttatg	63,8	endoglucanase
15	Q13_EG_fwd	gggtatctctcgagaaaagatgcagtcgtgctccgaaa	62,8	endoglucanase
16	Q13_EG_rev	caatgatgatgatgatgatggagtctctccttgacgaac	62,8	endoglucanase
17	Q16_EG_fwd	gggtatctctcgagaaaagagctatgctttgctgcattg	62,9	endoglucanase
18	Q16_EG_rev	caatgatgatgatgatgatgcaactgctgcttcacatatttg	62,9	endoglucanase
19	Q18_EG_fwd	gggtatctctcgagaaaagagccgtattgatgcttttcg	62,6	endoglucanase
20	Q18_EG_rev	caatgatgatgatgatgatgcaacatttcttttacaaactttcccc	62,6	endoglucanase
21	Q19_EG_fwd	gggtatctctcgagaaaagatgcactcaaaggtcgc	63,5	endoglucanase
22	Q19_EG_rev	caatgatgatgatgatgatgggcaacatcatgccaccg	63,5	endoglucanase
23	Q21_EG_fwd	gggtatctctcgagaaaagactggcagcaagctgctg	63,9	endoglucanase
24	Q21_EG_rev	caatgatgatgatgatgatggagattatcctttacaaatgctccc	63,9	endoglucanase
25	Q22_EG_fwd	gggtatctctcgagaaaagatgctcacagcaggctc	62,1	endoglucanase
26	Q22_EG_rev	caatgatgatgatgatgatgcagctgttcctttacaaacc	62,1	endoglucanase
27	Q25_GH5_fwd	gggtatctctcgagaaaagatgcacaaccggatcc	62,9	endoglucanase
28	Q25_GH5_rev	caatgatgatgatgatgatgcaatgccttcttctgcagg	62,9	endoglucanase
29	Q26_MrS_fwd	gggtatctctcgagaaaagagaaaatccggtagtgcgc	63	endoglucanase

30	Q26_MrS_rev	caatgatgatgatgatgatgcttcgcttttccctggat	63	endoglucanase
31	Q27_GH5_fwd	gggtatctctcgagaaaagatgtgggagcacaggttc	63,8	endoglucanase
32	Q27_GH5_rev	caatgatgatgatgatgatgtttcataagcaaagagttgatgtagc	63,8	endoglucanase
33	Q28_GH5_fwd	gggtatctctcgagaaaagatgcgcttctacccaatc	62,8	endoglucanase
34	Q28_GH5_rev	caatgatgatgatgatgatgttctgcagcctttctttgc	62,8	endoglucanase
35	b1_fwd	gggtatctctcgagaaaagaaacggtgtgaagtggaccttg	65,2	β-glucosidase
36	b1_rev	caatgatgatgatgatgatgacgggcatacttccttacac	65,2	β-glucosidase
37	b2_fwd	gggtatctctcgagaaaagaccacagctgacaaaagacaacatc	64,2	β-glucosidase
38	b2_rev	caatgatgatgatgatgatgtttcttgagagaaagttcgtttattgc	64,2	β-glucosidase
39	b3_fwd	gggtatctctcgagaaaagatgcacccagccgaaattcg	66,3	β-glucosidase
40	b3_rev	caatgatgatgatgatgatgattcaacatgaaatttaccattgtcttgacatc	66,3	β-glucosidase
41	b5_fwd	gggtatctctcgagaaaagaatcaaatatctttaccgactcctgc	65,1	β-glucosidase
42	b5_rev	caatgatgatgatgatgatgtttaacctcaaattcgacaggcttg	65,1	β-glucosidase
43	b6_fwd	gggtatctctcgagaaaagatgcaactgcaattcagtcgagc	65,2	β-glucosidase
44	b6_rev	caatgatgatgatgatgatgatcaatatttggatcgtcccagac	65,2	β-glucosidase
45	b7_fwd	gggtatctctcgagaaaagaaatctgtatccttatgaaaacgagcatattg	65,4	β-glucosidase
46	b7_rev	caatgatgatgatgatgatggctcctttccgcatttgtaagcttg	65,4	β-glucosidase
47	b8_fwd	gggtatctctcgagaaaagagatattaaatcaattatcaaaaatcttaccctcgaac	64,5	β-glucosidase
48	b8_rev	caatgatgatgatgatgatgaagttgtttttcgaatttagaatttgcgctaagg	64,5	β-glucosidase
49	b11_fwd	gggtatctctcgagaaaagacaggctccgcaacccg	66,6	β-glucosidase
50	b11_rev	caatgatgatgatgatgatggagaacttcgaaggctgtgg	66,6	β-glucosidase
51	b25_fwd	gggtatctctcgagaaaagatgcagtcagccgaaatttgacg	66,4	β-glucosidase
52	b25_rev	caatgatgatgatgatgatgtttaagttcgaatgaagcttccgc	66,4	β-glucosidase
53	b35_fwd	gggtatctctcgagaaaagaactaaccagcctgcctacaaag	64	β-glucosidase
54	b35_rev	caatgatgatgatgatgatgttgcaatgcaatggtttttgc	64	β-glucosidase

Table S3 List of GenBank accession numbers of the expressed sequences

BankIt3039195	Endoglucanase_3	PX898737
BankIt3039195	Endoglucanase_4	PX898738
BankIt3039195	Endoglucanase_5	PX898739
BankIt3039195	Endoglucanase_7	PX898740
BankIt3039195	Endoglucanase_11	PX898741
BankIt3039195	Endoglucanase_12	PX898742
BankIt3039195	Endoglucanase_13	PX898743
BankIt3039195	Endoglucanase_16	PX898744
BankIt3039195	Endoglucanase_18	PX898745
BankIt3039195	Endoglucanase_19	PX898746
BankIt3039195	Endoglucanase_21	PX898747
BankIt3039195	Endoglucanase_22	PX898748
BankIt3039195	Endoglucanase_25	PX898749
BankIt3039195	Endoglucanase_26	PX898750
BankIt3039195	Endoglucanase_27	PX898751
BankIt3039195	Endoglucanase_28	PX898752
BankIt3039853	beta-glucosidase_1	PX898753
BankIt3039853	beta-glucosidase_2	PX898754
BankIt3039853	beta-glucosidase_3	PX898755
BankIt3039853	beta-glucosidase_5	PX898756
BankIt3039853	beta-glucosidase_6	PX898757
BankIt3039853	beta-glucosidase_7	PX898758
BankIt3039853	beta-glucosidase_8	PX898759
BankIt3039853	beta-glucosidase_11	PX898760
BankIt3039853	beta-glucosidase_25	PX898761
BankIt3039853	beta-glucosidase_35	PX898762

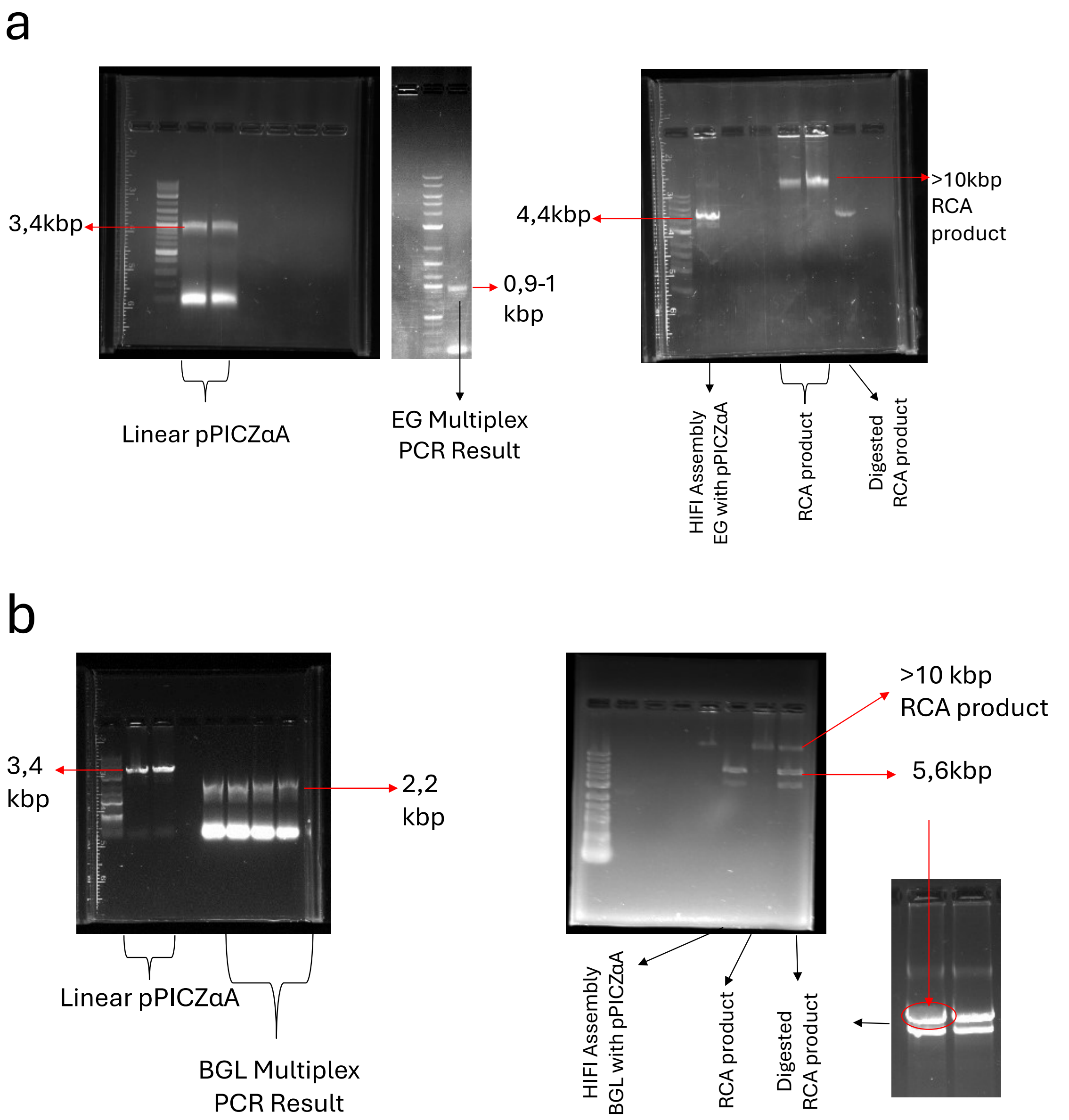

Fig. S1 Electrophoresis Results of Amplified Cellulases by Multiplex PCR, HIFI Assembly Cellulases to pPICZαA, and RCA (a) endoglucanase (b) β-glucosidase

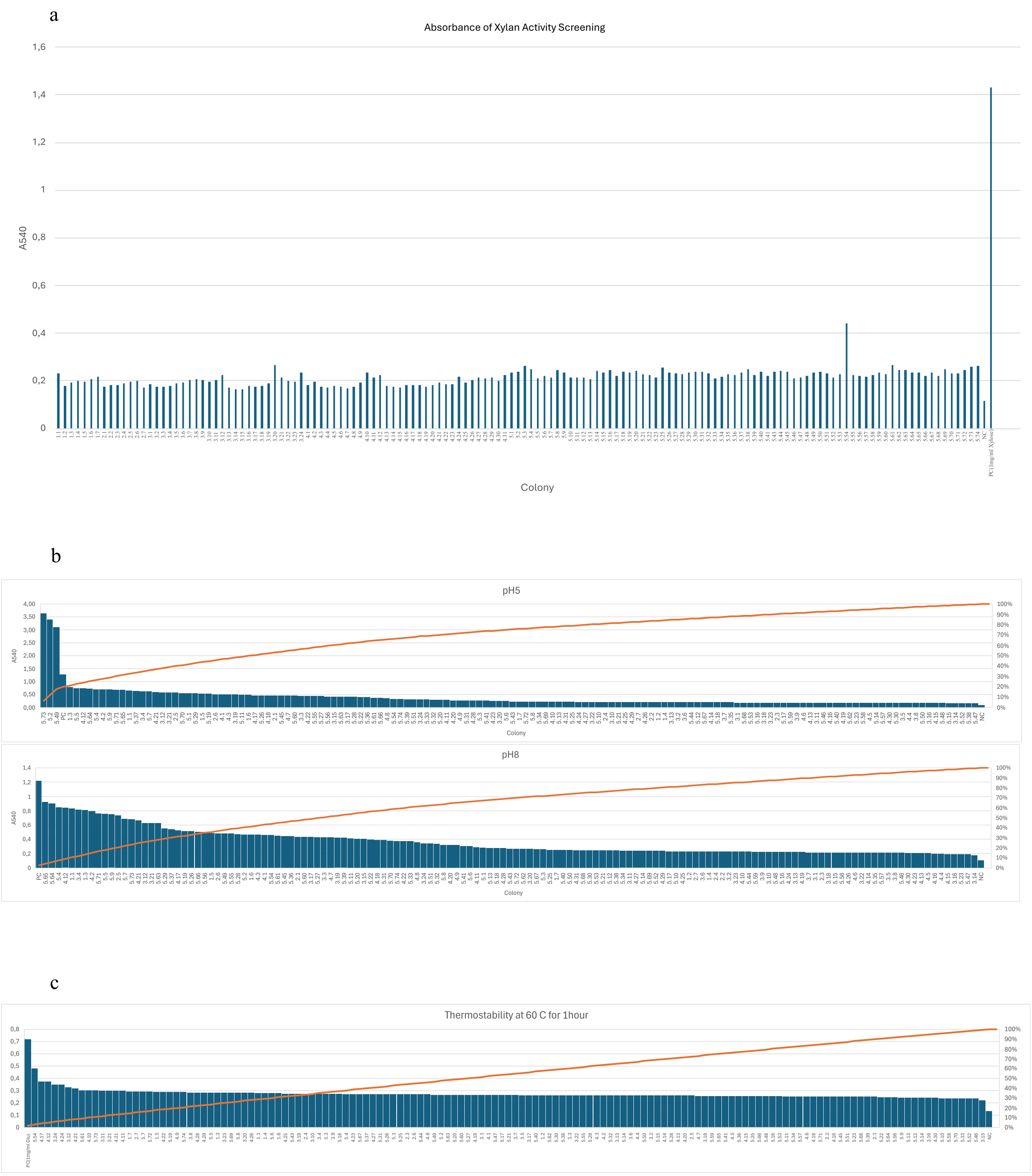

Fig. S2 Screening of colonies for (a) hydrolysis of xylan, (b) PH acid/alkaline and (c) thermostability. NC: negative control, PC: positive control
